# Influenza A(H1N1)pdm09 virus and asthma

**DOI:** 10.3389/fmicb.2013.00307

**Published:** 2013-10-14

**Authors:** Masatsugu Obuchi, Yuichi Adachi, Takenori Takizawa, Tetsutaro Sata

**Affiliations:** ^1^Department of Virology, Toyama Institute of HealthToyama, Japan; ^2^Department of Pediatrics, Faculty of Medicine, University of ToyamaToyama, Japan

**Keywords:** asthma, pandemic influenza, influenza A(H1N1)pdm09 virus, vaccine, antiviral drug

## Abstract

Respiratory viral infection is a major cause of asthma exacerbations in both children and adults. Among the respiratory viruses, influenza virus is a particularly important pathogen due to its enormous morbidity and mortality in annual epidemics. The swine-origin influenza A virus, designated as A(H1N1)pdm09, emerged in the spring of 2009 and caused the first influenza pandemic in the 21st century. With the emergence of the novel A(H1N1)pdm09 virus, numerous epidemiologic studies detected asthma as a frequent comorbid condition in patients infected with this virus. Here we review recent reports regarding asthma in patients infected with influenza A(H1N1)pdm09 virus, and we discuss the utility of influenza vaccines and antivirals.

## INTRODUCTION

Asthma is a chronic airway disease with the symptoms of repetitive cough, wheezing and dyspnea, with reversible airway narrowing accompanied by airway hyper-responsiveness (Ohta et al., 2011). It is estimated that worldwide, approximately 300 million people including both children and adults have asthma (Masoli et al., 2004). Inhaled irritants, inhaled allergens, and microorganism infections of the respiratory tract are common causes of asthma exacerbations. Respiratory viral infection is closely associated with asthma (Jacoby, 2002; Papadopoulos et al., 2011). Human rhinovirus (HRV) is the most common virus in asthmatics of all ages (Papadopoulos et al., 2011). Respiratory syncytial virus and enterovirus are also frequently detected in infants, whereas influenza virus seems to induce severe exacerbations, mostly in adults (Papadopoulos et al., 2011).

Influenza virus causes influenza characterized by a sudden onset of high fever and respiratory symptoms such as cough, sore throat and coryza, as well as systemic symptoms such as headache, muscle ache and fatigue. Influenza epidemics occur yearly during the autumn and winter in temperate regions, whereas the disease patterns in tropical and subtropical regions are less well established (World Health Organization, 2009). Annual epidemics result in approximately three to five million cases of severe illness and approximately 250,000 to 500,000 deaths, which occur mostly among people age 65 or older (World Health Organization, 2009).

## INFLUENZA A(H1N1)pdm09 VIRUS

Influenza A viruses can be subtyped according to their two major surface glycoproteins, hemagglutinin (HA) and neuraminidase (NA). Currently, there are 16 subtypes of HA (H1–H16) and nine subtypes of NA (N1–N9), and all have been found in wild aquatic birds, which are the natural reservoir of influenza A viruses. Only two subtypes of these viruses (H1N1 and H3N2) are currently circulating in humans, as seasonal influenza. Influenza A viruses have negative-sense, single-stranded, and eight-segmented RNAs as the genome (Lamb and Choppin, 1983). It is known that simultaneous infection of a single cell by two distinct influenza A viruses can lead to gene reassortment (Hause et al., 2012), which results in the generation of a novel influenza virus strain. It is believed that most human pandemic influenza A viruses arose in this manner.

In March and early April 2009, a novel swine-origin influenza A(H1N1) virus, designated as A(H1N1)pdm09, emerged in Mexico and the United States (Centers for Disease Control and Prevention, 2009) and rapidly spread worldwide. Genetic and evolutionary analyses revealed that this pandemic virus contains a combination of gene segments which had not been reported previously in swine or human influenza viruses in any part of the world. In the late 1990s, reassortment among North American avian (unknown subtype), human A(H3N2), and classical swine A(H1N1) viruses resulted in triple reassortant swine A(H3N2) and A(H1N2) viruses.

**FIGURE 1:**
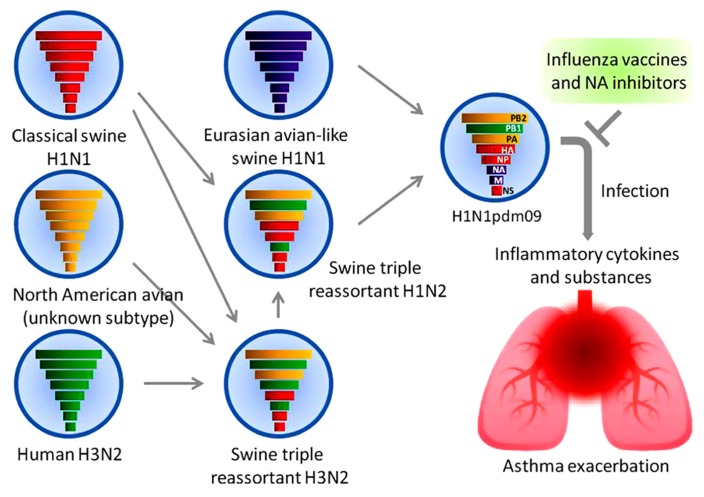
**A(H1N1)pdm09 virus and asthma.** The reassortment of a triple reassortant swine A(H1N2) virus with a Eurasian avian-like swine A(H1N1) virus resulted in the pandemic A(H1N1) 2009 virus. The colored solid rods represent the gene segments as follows. Classical swine A(H1N1) virus: red. North American avian virus: yellow. Human A(H3N2) virus: green. Eurasian avian-like swine A(H1N1) virus: purple. Airway inflammation induced by the viral infection causes an exacerbation of asthma. Early treatment with antiviral drugs and vaccination represents the mainstay of management.

A triple reassortant swine A(H1N2) virus then reassorted with a Eurasian avian-like swine A(H1N1) virus, resulting in A(H1N1)pdm09 virus (Garten et al., 2009; Smith et al., 2009; **Figure 1**). The polymerase basic 2 (PB2) and polymerase acidic (PA) gene segments were derived from the avian virus lineage, whereas the polymerase basic 1 (PB1) gene segment was from human A(H3N2) virus. The HA, nucleoprotein (NP), and non-structural protein (NS) gene segments were from classical swine A(H1N1) virus. The NA and matrix (M) gene segments were from the Eurasian avian-like swine A(H1N1) virus.

A(H1N1)pdm09 virus has none of the known hallmarks of virulent influenza viruses such as highly pathogenic avian A(H5) and A(H7) viruses, except for an amino acid substitution of aspartic acid by glycine at position 222 (D222G) in the HA, which was observed in severe and fatal cases with high frequency. The D222G substitution changes the receptor binding specificity of the virus from α2–6 (mammalian type) to α2–3 (avian type) sialylated glycans (Puzelli et al., 2010; World Health Organization, 2010; Belser et al., 2011). This amino acid substitution may result in a more efficient infection of human alveolar type II pneumocytes, which express avian type receptors, reducing the availability of progenitor cells for essential lung functions and thus leading to severe pulmonary impairment.

We recently reported that A(H1N1)pdm09 viral isolates derived from fatal cases manifested sporadic amino acid changes in the PB2 and PA proteins (which are subunits of viral RNA polymerase) more frequently than those derived from mild cases (Obuchi et al., 2012). More recently, reassortant viruses generated by reverse genetics have shown that lysine or isoleucine at position 340 or 649 of the PB2, respectively, and threonine at position 667 of the PB2 also contribute to virulence in a mouse model (Uraki et al., 2013). Further studies are needed to elucidate the role of the viral RNA polymerase of A(H1N1)pdm09 virus as a virulence factor.

## A(H1N1)pdm09 VIRAL INFECTION AND ASTHMA

Widespread activity of pandemic A(H1N1) 2009 occurred and reached its peak a couple of months earlier than the usual seasonal influenza in the northern hemisphere, from April 2009 to January 2010 (Amato-Gauci et al., 2011; Jhung et al., 2011). The A(H1N1)pdm09 viral infection was considered a mild disease, similar to seasonal influenza. However, many severe and fatal cases were observed not only in the high-risk groups, but also among healthy children and young adults during the pandemic waves (Athanasiou et al., 2010; Reichert et al., 2010).

Asthma was one of the most common underlying medical conditions among patients hospitalized with A(H1N1)pdm09 viral infection in 2009 worldwide (Jain et al., 2009; Van Kerkhove et al., 2011). Kloepfer et al. (2012) reported that children with asthma had increased susceptibility to A(H1N1)pdm09 viral infection. They collected weekly nasal samples from 161 children (95 with asthma and 66 without asthma) between September 5 and October 24, 2009, and a total of 346 viral infections were detected. The majority were HRV (62%), followed by enterovirus (12%), A(H1N1)pdm09 virus (10%), adenovirus (2%), and others. Overall, 34% of the children were infected with A(H1N1)pdm09 virus during the study period. The incidence of A(H1N1)pdm09 viral infection was significantly higher in the children with asthma (41%) than in the children without asthma (24%), whereas the incidences of HRV (95% each) and the other viral infections (47% vs. 41%) were similar.

A Canadian group reported that children admitted to a hospital with A(H1N1)pdm09 viral infection tended to have pre-existing asthma to a greater extent compared to the children with seasonal influenza A viral infection (15% vs. 5%), although there was no significant difference in the severity of pre-existing asthma between the groups of children with these infections (Morris et al., 2012). An age-matched control study in Hong Kong demonstrated that hospitalized children with A(H1N1)pdm09 viral infection were more susceptible to asthma exacerbations compared to seasonal A(H1N1) (8.1 vs. 1.0%) or A(H3N2) (8.1 vs. 1.0%) viral infection (Chiu et al., 2011). A Japanese group reported similar findings (Hasegawa et al., 2011). It seems likely that A(H1N1)pdm09 viral infection rather than A(H1N1) or A(H3N2) viral infection may enhance the already elevated inflammatory response and worsen the symptoms in asthma. The underlying mechanisms of increased susceptibility to A(H1N1)pdm09 viral infection and the asthma exacerbation remain to be explored.

In contrast, it is not clear whether A(H1N1)pdm09 viral infection can frequently cause the development of asthma compared to seasonal A(H1N1) or A(H3N3) viral infection. The study by Hasegawa et al. (2011) mentioned above showed that 7 (31.8%) of 22 asthmatic children with A(H1N1)pdm09 viral infection admitted to a hospital between October and December 2009 were not previously diagnosed with asthma. The sample size of that study is small, and thus a larger patient population must be studied.

Influenza A viral infection induces the production of interleukin 1 beta (IL-1β), IL-6, IL-8, tumor necrosis factor-alpha (TNF)-α, histamine, protease, interferon (IFN)-α, and IFN-gamma (IFN-γ) from airway epithelial cells and other cells including peripheral blood basophils (Yamaya, 2012). These proinflammatory cytokines, monokines, and inflammatory substances may contribute to the development of airway inflammation, damaging the barrier function and leading to a subsequent asthma attack (**Figure 1**).

Camp et al. (2013) examined the phenotypic differences in virulence and immune response in A(H1N1)pdm09 virus isolates obtained from hospitalized patients with severe pneumonia. In that study, all viral isolates showed high similarity in nucleic acid sequences in viral gene and replication levels in nasal turbinates and lung, but the isolates’ virulence and host responses in mice varied. Proinflammatory cytokines such as IL-1β, TNF-α and a keratinocyte-derived chemokine (KC) were expressed early in mice infected with virulent isolates compared to avirulent isolates, including a vaccine strain of A(H1N1) virus in the 2008–2009 season, A/Brisbane/59/2007. In vitro experiments demonstrated that a virulent isolate – but not an avirulent isolate – was able to replicate productively in macrophages, suggesting that viral susceptibility to macrophages may be one of the key determinants of their pathogenicity (Camp et al., 2013).

## UTILITY OF INFLUENZA VACCINES AND ANTIVIRAL DRUGS IN PATIENTS WITH ASTHMA

Many respiratory viruses are associated with asthma exacerbations, among which the influenza virus is the only virus for which both vaccines and antiviral drugs are available (**Figure 1**). Two types of influenza vaccines are currently available; inactivated vaccine and live, attenuated vaccine. The live, attenuated nasal-spray influenza vaccine has been approved for use in the United States since 2003. However, it has not been recommended in high-risk groups including asthmatics because its safety is not fully demonstrated. The widespread use of inactivated influenza vaccines contain a trivalent mixture of strains of A(H1N1)pdm09, A(H3N2), and type B viruses likely to circulate during the next influenza season. Many studies indicated that no increase in asthma exacerbations was reported for both vaccinated children and adults (American Lung Association Asthma Clinical Research Centers, 2001; Kramarz et al., 2001; Hak et al., 2005).

A randomized, open-label study to investigate the safety and immunogenicity of two administrations of an unadjuvanted, inactivated A(H1N1)pdm09 virus vaccine was conducted in the United States (Busse et al., 2011). The results indicated that both the 15-μg (standard dose) and 30-μg vaccine doses generally provided excellent seroprotection against viral antigen 21 days after a single immunization in patients (12 to 79 years of age) with mild-to-moderate asthma. In patients with severe asthma, the response to the 15-μg dose was lower than that to the 30-μg dose. The authors of that study did not identify any safety concerns with the A(H1N1)pdm09 vaccine. Collectively, the findings described above indicate that inactivated influenza vaccines are well tolerated in patients with asthma.

Specific antiviral drugs against influenza viruses could be used for the treatment and prophylaxis for influenza. Based on their chemical properties and spectra of activity against influenza viruses, the drugs can be classified into two categories: the M2 ion channel inhibitors, i.e., adamantanes (amantadine and rimantadine) and the NA inhibitors, i.e., zanamivir, oseltamivir, peramivir, and laninamivir. Currently, the NA inhibitors are exclusively used for the treatment and prophylaxis of influenza because the circulating strains of A(H1N1)pdm09 and A(H3N2) viruses have a known amino acid substitution of serine by asparagines at position 31 in the M2 protein, which confers resistance to the adamantanes.

Few studies have examined the safety of NA inhibitors in patients with asthma. A double-blind, placebo-controlled crossover study indicated that zanamivir inhaled as a dry powder did not significantly affect the pulmonary function and airway responsiveness of subjects (19 to 49 years of age) with mild or moderate asthma (Cass et al., 2000). However, a number of studies suggested that the use of NA inhibitors was beneficial in hospitalized patients with A(H1N1)pdm09 viral infection, particularly when they are started within 48 h after the onset of illness (Domínguez-Cherit et al., 2009; Jain et al., 2009; Louie et al., 2010). Accordingly, WHO recommended that for patients at increased risk for severe or complicated illness, treatment with oseltamivir or zanamivir should be started as soon as possible after the onset of illness (Writing Committee of the WHO Consultation on Clinical Aspects of Pandemic (H1N1) 2009 influenza, 2010). Although A(H1N1)pdm09 virus resistance to NA inhibitors has been detected at very low frequency among circulating viral strains (World Health Organization, 2013b), there is concern about the recent report that oseltamivir-resistant A(H1N1)pdm09 viral mutants were detected in untreated patients and from a few clusters in some countries (Samson et al., 2013).

## CLOSING REMARKS

Epidemiological studies as described above demonstrated that A(H1N1)pdm09 viral infection is closely associated with asthma in both children and adults. Although A(H1N1)pdm09 virus has not shown a high mortality rate similar to that of the highly pathogenic avian influenza virus of the H5N1 subtype, patients with A(H1N1)pdm09 viral infection were more susceptible to asthma exacerbation compared to A(H1N1) or A(H3N2) viral infection. Detailed analyses of virus-host interactions are needed to elucidate the mechanism underlying A(H1N1)pdm09 viral infection-induced asthma.

Since March 31, 2013 when the public health authorities of China reported three cases of human infection with an avian influenza A(H7N9) virus, a total of 135 human cases including 44 fatal cases have been reported in China and Taiwan as of August 12, 2013 (World Health Organization, 2013a). The current avian influenza viral infections in humans present considerable pathogenic potential with high mortality rates, suggesting that the pandemic viruses, if they emerge in human beings, could also present high pathogenicity and result in an excessive number of deaths in high-risk groups, including asthmatics. It will therefore be important to make preparations for drugs and vaccines for anti-influenza treatments and the prophylaxis of influenza.

## Conflict of Interest Statement

The authors declare that the research was conducted in the absence of any commercial or financial relationships that could be construed as a potential conflict of interest.
